# Artificial Neural Networks for Pyrolysis, Thermal Analysis, and Thermokinetic Studies: The Status Quo

**DOI:** 10.3390/molecules26123727

**Published:** 2021-06-18

**Authors:** Nikita V. Muravyev, Giorgio Luciano, Heitor Luiz Ornaghi, Roman Svoboda, Sergey Vyazovkin

**Affiliations:** 1 N.N. Semenov Federal Research Center for Chemical Physics, Russian Academy of Sciences, 4 Kosygina Str., 119991 Moscow, Russia; 2 CNR, Istituto di Scienze e Tecnologie Chimiche “Giulio Natta”, Via De Marini 6, 16149 Genova, Italy; giorgio.luciano@ge.ismac.cnr.it; 3 Department of Materials, Federal University for Latin American Integration (UNILA), Silvio Américo Sasdelli Avenue, 1842, Foz do Iguaçu-Paraná 85866-000, Brazil; hlornagj@ucs.br; 4 Department of Physical Chemistry, Faculty of Chemical Technology, University of Pardubice, Studentská 95, CZ-53210 Pardubice, Czech Republic; roman.svoboda@upce.cz; 5 Department of Chemistry, University of Alabama at Birmingham, 901 S. 14th Street, Birmingham, AL 35294, USA

**Keywords:** artificial neural networks, conversion degree, kinetics, machine learning, pyrolysis, thermal analysis

## Abstract

Artificial neural networks (ANNs) are a method of machine learning (ML) that is now widely used in physics, chemistry, and material science. ANN can learn from data to identify nonlinear trends and give accurate predictions. ML methods, and ANNs in particular, have already demonstrated their worth in solving various chemical engineering problems, but applications in pyrolysis, thermal analysis, and, especially, thermokinetic studies are still in an initiatory stage. The present article gives a critical overview and summary of the available literature on applying ANNs in the field of pyrolysis, thermal analysis, and thermokinetic studies. More than 100 papers from these research areas are surveyed. Some approaches from the broad field of chemical engineering are discussed as the venues for possible transfer to the field of pyrolysis and thermal analysis studies in general. It is stressed that the current thermokinetic applications of ANNs are yet to evolve significantly to reach the capabilities of the existing isoconversional and model-fitting methods.

## 1. Introduction

Machine learning has found a number of applications in chemistry [1,2,3] and material science [4,5,6]. Among the supervised machine learning methods, the artificial neural networks (ANN) are most popular. Artificial neural networks are the models inspired by biological neural systems such as the human brain [7]. ANN represents the oriented graphs with nodes called by the same analogy with brain as “neurons”. The key features of the neural network are its topology (number of layers and neurons in it) and the strength of connections between the neurons (defined by mathematical weights). The benefits of ANN include the capability to approximate any continuous non-linear function, and high tolerance to noisy or missing data [8,9]. Thanks to these properties, the neural networks have found their use in vastly different fields, e.g., image classification [10], predicting combustion instability [11], or impact sensitivity of energetic materials [12]. Although ANNs have been extensively applied in chemical engineering since the 1990s [13,14,15], their usage in thermal analysis has commenced later [9,16], and most of the studies emerged only recently [17,18,19,20,21]. The present review aims to summarize such studies and to assess some future prospects.

Thermal analysis (TA) is defined as “the study of the relationship between a sample property and its temperature as the sample is heated or cooled in a controlled manner” [22]. In this review, we focus mainly on applications of neural networks to thermal analysis and thermokinetic studies. Additionally, as one can see from the literature survey, typical applications involve pyrolysis as a thermal process considered most commonly by researchers. To keep the scope of review concise, the works on prediction of yield of pyrolysis product (regarded as a sort of TA experiment) are included, while reports on the selectivity and kinetics of pyrolysis gas products quantified with ANNs [23,24,25] are not covered in this review.

The most common methods of thermal analysis are differential scanning calorimetry (DSC) and thermogravimetric analysis (TGA) normally applied under linear rising temperature or constant temperature conditions. DSC produces a differential signal, i.e., the heat flow data, that is generally assumed to be proportional to the conversion rate dα/dt. This assumption ignores the thermal inertia term in the heat flow that is a potential source of a systematic error in kinetic evaluations [26]. However, this error tends to be negligible when using smaller masses and slower heating rates [27]. In turn, TGA data, representing the mass change of the sample, is an integral signal, and its normalized change yields the conversion degree α. The resulting conversion degree plotted against temperature and time is also known as the transformation–time–temperature (TTT) dataset. With this data array, the thermokinetic analysis is then performed. The procedure relies on the following basic equation [28]:

(1) $$\frac{d\alpha }{dt}=k\left(T\right)f\left(\alpha \right),$$

The multiplicand in Equation (1), i.e., the rate constant, usually assumes an Arrhenius form $k\left(T\right)=Aexp\left(-E_{a}/RT\right)$ with *E_a_* being the activation energy and *A* the preexponential factor. The multiplier in Equation (1) is the reaction model. Various approaches have been proposed for thermokinetic analysis, the most effective being the model-fitting [29,30] and the isoconversional [31,32] techniques. The obtained kinetic parameters generally serve for obtaining mechanistic insights into the processes [33,34], or for predicting their thermal behavior [35].

**Figure 1:**
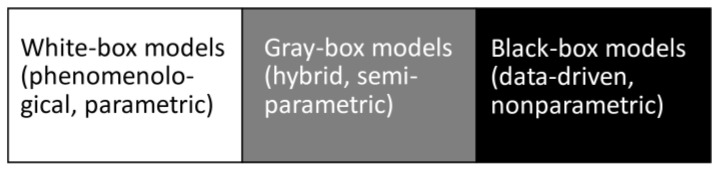
Three conceptual approaches to chemical engineering modeling.

From the conceptual point of view, the traditional thermokinetic analysis, i.e., Equation (1) with the kinetic triplet (*A*, *E_a_*, f(α)), is a mechanistic, phenomenological, or parametric modeling. The models of this type are known as white-box models, in contrast to the data-driven or nonparametric black-box modelling (Figure 1). The papers discussed in the present review are primarily concerned with the latter approach. To give a simple example of a black box model, let us imagine a neural network that is trained to output the conversion rate data once fed with temperature and time values. This ANN is readily applicable and does not require knowledge of the process kinetics. If carefully designed and properly trained, the network offers good descriptive accuracy within the domain of the supplied process data. Main limitations of the black-box models are the uncertain interpretation [36] and poor generalization ability [37]. To combine the benefits of white- and black-box approaches, hybrid semi-parametric modeling is proposed [38] (Figure 1). The application of hybrid models will be addressed in detail in Section 4 of this review. It is worth mentioning that some of the further-discussed ANNs are trained on synthetic data generated with Equation (1) or give as an output the kinetic parameters of Equation (1). Such models cannot be regarded as authentic black-box models because they either implicitly or explicitly utilize a phenomenological model.

**Figure 2:**
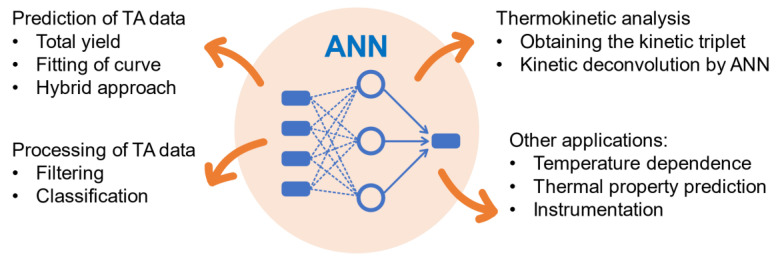
Logical scheme of the present review. Several aspects of using artificial neural networks (ANN) for thermal analysis (TA) studies are indicated to be discussed in main text.

The present review briefly surveys the conceptual details of ANNs, and summarizes their applications in pyrolysis, thermal analysis, and kinetic studies. A strong emphasis is put on the kinetic applications as this is a quickly growing field that needs special attention for proper development. Figure 2 schematically shows the blocks that represent major topics identified via analysis of the literature. The article is roughly structured according to these topics that are presented as the article sections. Note that the divisions between the sections are rather conditional and that the topics covered oftentimes overlap with other chemical engineering or chemometric problems. The review is intended to reflect the existing state of affairs in the area of the aforementioned applications and to provide certain critique, and to highlight some desired directions for future research.

## 2. Technical Details behind ANNs

Before discussing general aspects of the ANNs implementation, we should recognize that there is no single mathematical tool that resolves all TA-specific problems. ANN is only one of several machine learning methods and its accuracy for specific applications is hardly assessed a priori (this situation is known in mathematics as a “no free lunch” theorem [39]). However, for some applications the ANN model has been compared with other machine learning approaches, e.g., random forest [40], gradient boosting machine [40], and it has been found that ANN performs better.

Several types of ANNs are used in chemical engineering studies (e.g., see review by Ali et al. [41]). Nonetheless, almost all papers considered in the present review utilize the same type of ANN known as feed-forward neural network, which is briefly described below. More practical details are readily available from monographic sources [42,43].

**Figure 3:**
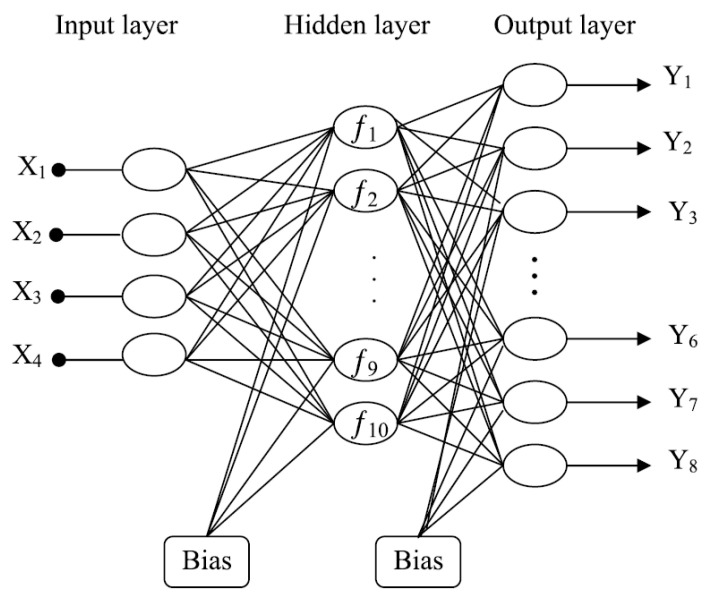
Scheme of the artificial neural network with indicated layers, weights, functions [51]. Reprinted with permission from Taylor & Francis.

ANN is a parallel processing system of interconnected computational elements, called neurons (Figure 3). The neurons are arranged in several layers—input, hidden, and outer layers. The topology of ANN is encoded numerically as follows, e.g., 25-15-10 denotes the neural network with 25 neurons in the input layer, 15 neurons in the hidden layer, and 10 neurons in the output layer. Note that most studies reviewed adopt the architecture with a single hidden layer, rarely two hidden layers are used [20,44], and only for exceptionally complex thermal profiles the authors has had to introduce even more hidden layers [45]. Connections between the neurons are displayed as lines in Figure 2, each connection has its own strength value called weight. Output of the neuron is determined by each layer’s transfer functions (activation function). While in most studies the hyperparameters (parameters used to control the learning process) and parameters of ANN are tuned manually, there are some techniques to make the procedure more rigorous and human-independent. In particular, the differential evolution algorithm can be utilized to optimize the number of hidden layers, number of neurons in it, weights, biases, and activation functions [46].

Let us consider ANN in more detail. The output of *i*-th neuron in (*L* + 1)-th layer is denoted as $y_{i}^{L+1}$. This value is comprised of a specific constant value called bias $b_{i}^{L+1}$, and *N*_L_ signals from previous layers, amplified or weakened by the corresponding coefficients, called weights $w_{ij}^{L+1}$ [19]:

(2) $$y_{i}^{L+1}=f\left(\sum_{j=1}^{N_{L}}w_{ij}^{L+1}y_{j}^{L}+b_{i}^{L+1}\right).$$

Among a broad variety of the activation functions [42,43], those of sigmoidal type $f\left(u\right)=Logsig\left(u\right)=1/\left(1+e^{-u}\right)$, symmetric sigmoidal form $f\left(u\right)=Tansig\left(u\right)=-1+2/\left(1+e^{-2u}\right)$ have been used most frequently. Thus, the ANN model transforms the input vector (x_1_, x_2_, …, x_i_) to the output vector (y_1_, y_2_, … y_j_). Before using, ANN is trained on the [(x_1_, y_1_), …, (x_n_, y_n_)] data, by modifying the weights of connections between the neurons to minimize the prediction error (supervised learning). Although more efficient and modern algorithms (e.g., Stochastic Gradient Descent or Adaptive Moment Estimation) are used broadly in ANN training, the application considered in the present review appear to be limited primarily to the Levenberg–Marquardt or error backpropagation algorithms. The key problem with the optimization is that it can converge to local minima. Some modifications for the common procedures are constantly proposed to improve the computational time and robustness. For example, ant colony optimization (ACO) algorithm is suggested [47] to improve the convergence of backpropagation technique, and it is shown that it slightly improves the performance of ANN [48]. To evaluate how well ANN predicts in the domains not used for training, the data are usually split into the training and validation sets. The split ratio should generally depend on the quality and structure of the data. However, our analysis of the literature concerned with the present review topic suggests that the typical ratio for sizes of the training and validation sets is ~70% and ~30%, respectively. The realistic confidence limits for ANN predictions can be generated as described elsewhere [49]. Ensemble learning techniques, like stacking of several ANNs, boosting or bagging, have been suggested as a viable methods to further improve the generalization ability [50].

Optimal ANN architecture is defined by learning quality and generalization ability. Both parameters rely on the data, which should be representative and big enough in size. However, as the studies considered below mostly involve the time-consuming and labor-intensive measurements, the size of available data is usually small, especially when compared with typical applications of machine learning. Just to give an example of typical number of learning data, 50,000 images are used to train ANN to recognize the handwritten numbers from 1 to 10 [52]. For the thermal behavior data, the amount of input data is defined by a particular experimental setup and sampling frequency, but usually is far less than the number above. To give an estimate of how much data is needed, we notice that the theoretical estimates suggest that the number of learning patterns necessary to attain the sufficient generalization ability should be ten times larger than the total number of weights in ANN [53,54]. The problem of sparse data can be alleviated by the generation of the artificial raw thermal analysis data [21], as discussed in the following section.

An important aspect of the application of ANNs to the TA-generated data is the representation of the input data. TA data used for thermokinetic analysis are usually in the form of several sets of three-dimensional data (conversion, temperature, time). These data should be transformed to decrease the dimensionality of the information being fed to the neurons of the input layer. The simplest way of doing this is to limit input to a single experiment and input one of the dependent parameters, e.g., time for isothermal runs [17], and train ANN to output the conversion data (note, that utilization of data corresponding only to a single run is not recommended from the kinetic point of view [28], although in some specific cases the approach could work). Raw thermal analysis signal can be considered as the output, while the temperature is the independent variable and each heating rate is considered separately [20,55]. A set of temperature values corresponding to the specific conversion degrees is used as inputs, and the single row of input vector is formed from several experiments performed at different heating rates [21,56]. This thin-out procedure should retain the kinetic information, i.e., have considerable number of points, and contain the regions with critical information [9,16] (peak, inflexion, etc.).

Some more advanced methods for mapping the multi-dimensional thermal analysis data for the input layer of neural network still need to be developed. Muravyev and Pivkina [21] propose using principal component analysis [57,58,59] or introducing additional hidden layers for this purpose. To the best of authors’ knowledge, no more advanced methods of introduction of TA data to ANN have been proposed so far. Possibly, some methods from other fields can be borrowed, e.g., that employed for spectral dimensionality reduction in spectroscopy and signal analysis [60].

Finally, reporting of the optimized architecture of the ANN model is strongly recommended. Only a few examples [19,61,62] are found in the reviewed literature that provide the network biases and weights. As a guiding principle, one must provide enough detail on the ANN architecture, so that others can reproduce the computations. This principle is commonly accepted in reporting experimental studies. It is only prudent to follow it in publishing ANN work.

## 3. Prediction of Conversion Data (Single Value, Whole Curve)

There is a number of papers [63,64,65,66] on the application of ANNs for obtaining the total conversion (e.g., mass loss by TGA) using experimental conditions as an input. One of the first papers on the topic has been by Carsky and Kuwornoo [67] who used ANN to predict the main quantities for coal pyrolysis: tar, volatiles, and char yields. Table 1 shows the input parameters, which those authors have compiled from the literature on pyrolysis of several varieties of coals in fluidized bed reactor, spouted bed reactor, hot-rod, and wire-mesh reactors (since the experiments comprise the heating and isothermal hold steps, we consider them as TA data). An important aspect of the study is that the input parameters are in most cases not distributed uniformly or continuously. Thus, the authors have used, additionally, the fuzzy modeling to transform the particle holding time. For other variables, e.g., particle size, a qualitative classification (fine, coarse) was used instead of unevenly distributed numerical values. The optimized neural network (47-16-3) has described well the experimental tar, volatiles, and char yields, as is shown by root mean square errors of 4.2, 4.9, and 6.4%, respectively.

**Figure 4:**
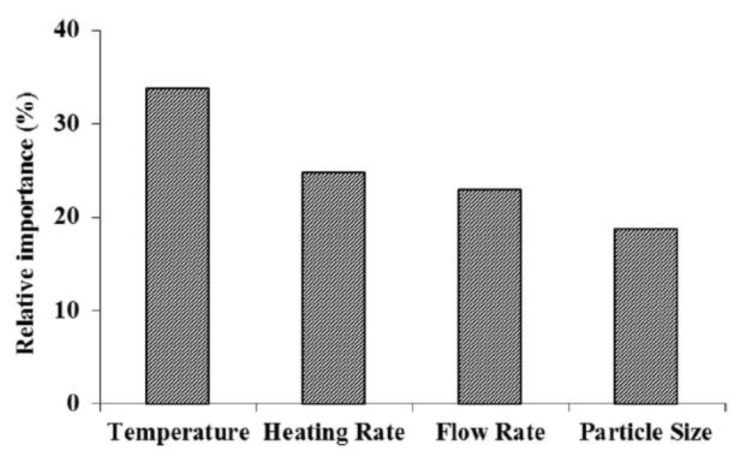
The example of relative importance analysis for input descriptors of trained neural network model [63]. Reprinted by permission from Springer Nature.

Thus, originally, this approach has been proposed to derive a single final value of conversion. In more recent studies, the idea has been extended to employ ANNs for predicting the conversion values at various moments, i.e., a TA signal itself. Such studies differ drastically in experimental systems (*Tithonia diversifolia* weed biomass [63], low-density polyethylene [64], high-density polyethylene [68], safflower seed press cake [65], durian rinds [69], rape straw [70], coal gangue and peanut shell [71], pet coke [72], sewage sludge and peanut shell [73], sewage sludge and coffee grounds [74], vegetable fibers [75], rice husk and sewage sludge [45], sludge, watermelon rind, corncob, and eucalyptus [76], *Sargassum* sp. seaweed [77], cotton cocoon shell, tea waste, and olive husk [66], mechanoactivated coals [78], cattle manure [79], lignocellulosic forest residue and olive oil residue [80], cotton cocoon shell, fabricated tea waste, olive husk, and hazelnut shell [81]) and in some minor details, but the general concept remains the same. Thus, we illustrate it with the study by Kataki et al. [63], who used a neural network model (4-14-1) to predict the product yield for the pyrolysis of dried weed biomass. The four input parameters have been the pyrolysis temperature (during the 30 min isothermal segment), heating rate used to attain this temperature, nitrogen flow rate, and particle size of the specimen. Thirty pyrolysis experiments with varying experimental conditions have been designed and performed, and the target output (yield of the bio-oil) has been determined. The optimized ANN has demonstrated a better accuracy than the response surface method used as an alternative. Finally, ANN has been used to design the experimental conditions for maximizing the output quantity, and the result has agreed closely with the neural network prediction. Although the inner relationships of ANN are not known, some insights can be gained by looking at the relative importance of the input descriptors. Figure 4 shows that the pyrolysis temperature is the main factor (more than 30% of relative importance) followed by heating rate (25%), flow rate (22%), and particle size (18%). The discussed approach can be considered as a sort of experimental design and can be definitely used for industrial thermal processes to optimize the single target property (e.g., yield of product).

**Figure 5:**
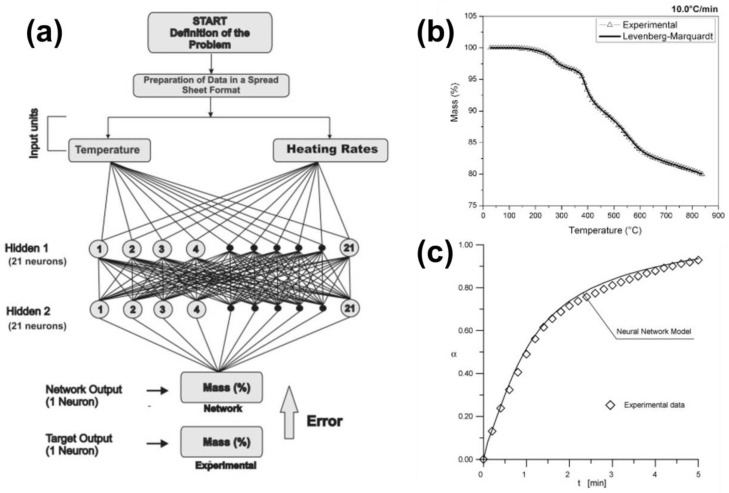
Typical approach to thermal signal fit with ANN model: (**a**) the workflow [20], and examples of use (**b**,**c**). Nonisothermal data (**b**) corresponds to the pyrolysis of carbon reinforced carbon composite (heating rate of 10 K min^−1^) [20]. Isothermal data (**c**) represents the iron oxide reduction (*T* = 880 °C, environmental gas 90% N_2_ + 10% CO) [19]. Reprinted with permission from Elsevier.

The idea from the above-mentioned studies can be expanded further. ANN can be trained to predict the conversion degree (or the primary TA signal) based on temperature and time. This approach can be classified as a nonlinear fit of the TA data, because no extrapolation is performed, and all data are within the experimental domain space. The graphical representation of the workflow from the study by Bezerra et al. [20] is shown in Figure 5a. It is rather a general approach that predicts the thermal signal from a principal descriptor (temperature) and another parameter, which defines the specific nonisothermal measurement, the heating rate. Figure 5b,c show the good quality fit via neural network models applied to complex thermally-induced processes. Figure 5b illustrates the experimental TGA signal for nonisothermal pyrolysis of a carbon reinforced fiber composite along with its fit via the ANN model (2-21-21-1). Figure 5c represents the isothermal data for iron oxide reduction process [19] fitted with a neural network (3-20-1). In both cases, ANN offers an excellent fit of experimental data.

As one may notice, the ANN used to obtain the data shown in Figure 5b contains two hidden layers, whereas the one used to fit the data from Figure 5c contains only a single hidden layer of neurons. The selection of complexity of the ANN topology is, thus, forced by the data. This observation is in line with the findings by Cepeliogullar et al. [44]. They have studied the pyrolysis of refuse-derived fuel and analyzed in detail the impact of the ANN architecture parameters. Due to the complex thermal behavior of the investigated highly heterogeneous samples, only after introduction of the second hidden layer of neurons an adequate fitting quality is achieved. In addition to the number of neurons in both hidden layers, the authors considered various activation functions. For the optimal neural network, the prediction ability was tested by comparison with direct experiment not used in the model development. The predicted mass loss curve for heating rate of 25 K min^−1^ closely agrees with the experiment. Note, however, that ANN has been trained with data acquired at 5, 10, 15, 20, 30, 40, and 50 K min^−1^, thus, this prediction is still within that domain space and it is, in fact, a sort of interpolation.

Burgaz et al. [82] have applied the neural network models to directly predict several thermal analysis signals (DSC, TGA, and dynamic mechanical analysis, DMA) for poly(ethylene oxide)/clay nanocomposites. The input parameters are the amount of clay in the sample and temperature, other details are summarized in Table 2. Authors note that the amount of data necessary to attain good performance is signal-dependent, i.e., it is smaller for DMA than for the TGA and DSC signals. The higher complexity of the mass loss and heat flow signals also forces the use of two hidden layers instead of only one for DMA.

From the research discussed in this section, we can conclude that ANNs can successfully fit various thermal signals. The topology of the ANN model and other parameters depend on the amount of learning data, the selected input parameters, but most profoundly on the inherent complexity of data. The examples available so far do not include some testing on distant extrapolation accuracy of the neural networks output. Research in this direction would certainly be of value. In all, due to its excellent interpolation accuracy, this particular approach (conversion prediction from the experimental conditions) already has a plenty of industrial applications.

## 4. Advancements in Prediction of Conversion Data with ANNs (Moving Window, Hybrid Models)

**Figure 6:**
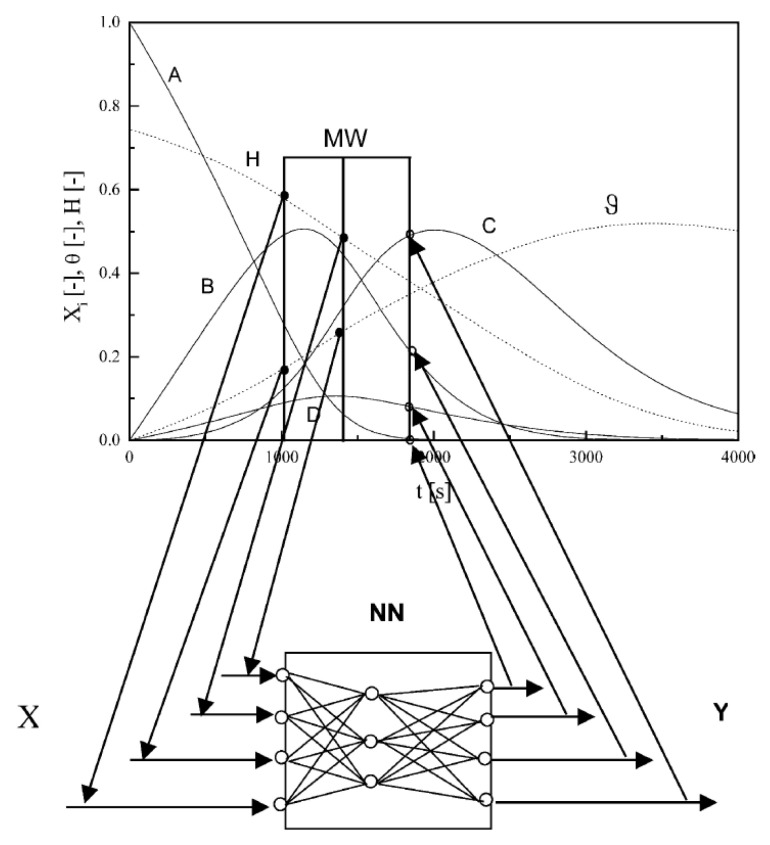
Modeling of the reactor by neural networks with moving time window [54]. X is the vector of input data, Y is vector of output data. Reprinted with permission from Elsevier.

It appears that chemical engineering has several approaches to the ANN applications that can be transferred to pyrolysis and thermal analysis problems. Note that publications discussed in the previous section do not use the conversion data as input, but using such input in ANNs with moving time window is highly beneficial. Figure 6 illustrates schematically the concept [54]: the input data include the temperature (pressure) data and the concentration values from previous time steps and the output data are the concentration of the reaction products at this particular time period. The depth of the time shift and the sampling time become important parameters of the model. The neural model with moving window outperforms the traditional neural network as shown on the data for hydrogenation of 2,4-dinitrotoluene in stirred tank reactor [54]. The main drawback of this kind of ANNs is the increased number of parameters that necessitates large quantities of input data and results in considerable computational times [83].

The utilization of neural networks primarily for prediction of the conversion profiles can be considered as a sort of black-box approach. It has no need of knowledge of the process fundamentals. However, alternatively to this fully black-box approach, the hybrid approach is proposed [38], being a combination of traditional modeling with description by ANN of the unknown part of the process (e.g., the reaction kinetics). It is deemed that the latter technique is beneficial as it affords some degree of the generalization of the results [54]. We are not aware of using this approach to the specific applications (i.e., to pyrolysis, thermal analysis, and thermokinetic studies) considered in the present review, thus, we make an excursus in chemical engineering to introduce the basic idea of the method.

To exemplify the hybrid approach, let us consider the study of heterogeneous oxidation of 2-octanol with nitric acid by Molga et al. [84] The chemical reaction was performed in the reaction calorimeter (RC1, Mettler-Toledo) and during the experiment, a small amount of samples were extracted and analyzed chemically. The adopted reaction mechanism comprises the oxidation of 2-octanol (A) to 2-octanone (P) by action of nitrosonium ion (B), and the subsequent oxidation with formation of carboxylic acids (X):

(3) A+B→P+2B, P+B→X.

**Figure 7:**
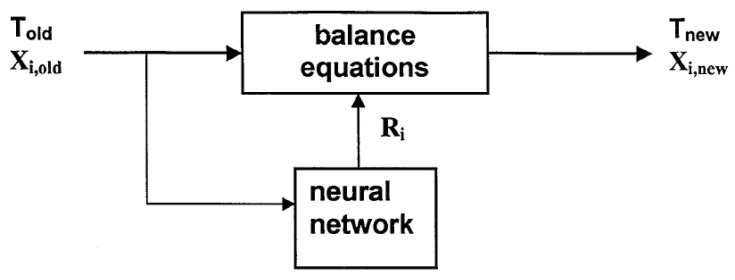
Schematic diagram of the hybrid model of chemical reactor [84]. Reprinted with permission from Elsevier.

The neural network works here as a kinetic model: it gets the temperature and concentrations of *A*, *P*, *X*, *B* as inputs and outputs the reactions rates for two reactions, *R*_1_, *R*_2_. ANN is then used in combination with traditional mass balance and heat balance equations, e.g.:

(4) $$\frac{dn_{P}}{dt}=\left(R_{1}-R_{2}\right)\left(n_{A,0}+n_{N,0}\right),$$

(*n*_i_ is molar amount of product *i* in the system, subscript *N* stands for the nitric acid, a source of nitrosonium ion). That is, the hybrid model is built, where the system of equations is solved with the kinetic data coming from the trained ANN (Figure 7).

This hybrid approach is well developed in chemical engineering [85], but it has not been yet applied systematically to the problems considered in the present review. One of the examples of its application is the study by Guo et al. [86], where the hybrid model was built for gas production rates in the course of coal pyrolysis. More research with this approach would be welcome. For example, it should be useful for purposes of thermal behavior prediction [35]. The conversion rate term will be supplied by ANN from input *T*_i_, α_i-1_ and then the output dα/dt|_i_ value will enter the conventional phenomenological model, thermal balance equations for storage condition [87]:

(5) $$m_{s}c_{p,s}\frac{dT_{s}}{dt}+m_{s}c_{p,c}\frac{dT_{c}}{dt}=US\left(T_{env}-T_{c}\right)+m_{s}\Delta H_{d}\frac{d\alpha }{dt},$$

where Δ*H_d_* is the enthalpy of decomposition reaction, *U*—the heat transfer coefficient, *S*—the area of a contact surface between sample and container, *c_p_*, *m*, and *T* stand for the isobaric heat capacity, mass, and temperature, correspondingly. The subscripts *s, c, env*. refer to the sample, container, and environment parameters, respectively.

There are other ways of combining traditional modeling with that by neural network [85,88]. An example is a study by Curteanu et al. [46] where the conversion and molar weights (α, MW) for the free radical polymerization of styrene are obtained by various models as the functions of time, temperature, and concentration of the initiator. Specifically, the authors consider four situations: (1) serial, the process parameters are an input to the traditional model and its output (α, MW) is sent to the ANN model that gives the final values of (α, MW); (2) parallel, both, the traditional model and ANN are fed with the process parameters and give the (α, MW) values, these values are averaged to give the final result; (3) serial-parallel, the ANN corrects the output by traditional model; (4) the traditional model output is used up to the time of gel effect appearance, then the output of the ANN model takes over. The latter situation shows the best accuracy of prediction, and the approach is probably transferable to other processes with the change of reaction mechanism.

## 5. Processing of the Raw Thermal Analysis Data (Filtering, Classification)

First, we consider possible applications of ANNs for treatment and analysis of the raw thermal analysis data. Sbirrazzuoli et al. [9,16] have proposed using the neural network models for filtering and deconvolution of the DSC data. Caloric thermoanalytical signals have been generated by the Joule effect. The respective electric pulse has been programmed to certain thermodynamic and kinetic parameters. ANN has been trained on the resulting DSC data setting as target values the expected parameters. In this manner, the transfer function of the calorimeter can be obtained and used for deconvolution of DSC data. Moreover, the results have shown high tolerance of ANNs to a noise introduced in the simulated DSC data.

**Figure 8:**
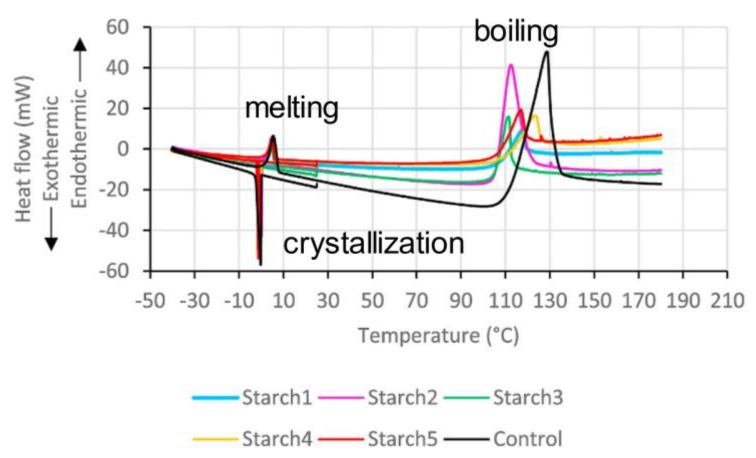
DSC signals for milk samples adulterated with various amount of starch [40]. Thermal processes that take place during cooling and subsequent heating are indicated. Reprinted with permission from Elsevier.

Another important aspect of the ANNs application to the raw TA data is their ability to recognize some regularities in the data. Recently, Cruz et al. [40] have proposed to employ ANNs for detecting adulteration in milk. That is, the DSC curves for samples with varying content of additives (starch, formaldehyde, whey, urea) show certain differences, and the general idea is to train the neural network to detect these undesired additives by analyzing the DSC data. The DSC measurements have comprised the cooling stage (from 25 °C to −40 °C at 10 K min^−1^ rate) followed by the heating step up to 180 °C at 10 K min^−1^. Typical DSC curves obtained in this manner are shown in Figure 8. The observed thermal events were analyzed in terms of the onset and peak temperatures and heat effects. Table 3 compiles the results for pure milk (the control sample) and milk with different amount of the starch additive, the subscripts *c*, *m*, and *b* correspond to the crystallization, melting, and boiling processes.

As seen from Table 3, the measured quantities are all altered by the additive presence, and the main purpose of that study is to train the neural network to identify the adulterated sample. This is a chemometric problem of classification, i.e., ANN should output 1 for the correct additive type and 0 for another additive types. The ANN (9-3-5) has been used. The input data are nine measured thermal properties (first column in Table 1), and the outputs are five sample classes (control, starch, formaldehyde, whey, urea). Apart from ANN, other machine learning methods have been tested, namely, the random forest and gradient boosting machine (GBM). Excellent prediction capacity (100%) is accomplished for ANN and GBM methods. Interestingly, these two top-performing methods reveal different values of the input variables importance, e.g., the onset of melting *T*_om_ is deemed not important by ANN (only 14%), whereas it is one of most important predictors by GBM (84%). Overall, the application of ANN for preliminary analysis of TA results is very promising. This example of the classification problem can be easily extended to other problems of analytical and industrial relevance.

**Figure 9:**
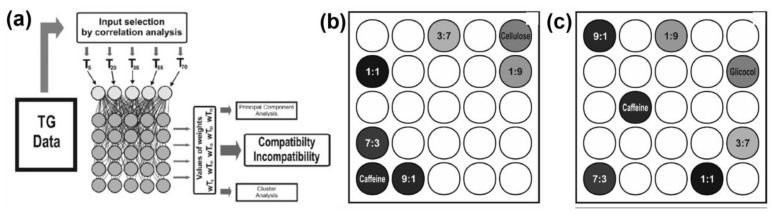
ANN application in the compatibility assessment [89]: (**a**) Schematic of the proposed workflow and mapping with ANN of caffeine mixtures with cellulose (**b**) and glycine (**c**), mixture ratios are indicated, i.e., 9:1, 7:3, 1:1, 3:7, 1:9. Reprinted with permission from Elsevier.

Wesolowski et al. [89] have suggested utilizing neural networks for compatibility assessment. This study appears to be the only example of the Kohonen network [90] (also known as self-organizing map, SOM) being applied directly to the TA data. In short, this type of network has no hidden layer and serves to map the input data into a two-dimensional grid. SOMs differ also in the training method; they apply unsupervised competitive learning. Figure 9b,c gives examples of the output topological maps. In their work [89], the authors looked at the thermogravimetric profiles of caffeine mixtures with several excipients, e.g., cellulose and glycine. First, the correlation matrix between the temperatures at certain mass loss values is built. Several temperature values at specific mass loss percentages (*T*_5_, *T*_20_, *T*_35_, *T*_55_, *T*_70_) that show strong correlation are selected and serve as inputs in SOM (Figure 9a). The optimal map size was found to be 5 × 5. Figure 9b,c shows the results of calculations for two typical mixtures. The highlighted neuron for the particular sample is the one that satisfies best the selection criteria. The interpretation of SOM maps is based on the assumption that the samples with close compositions should nest in allied regions. Thus, for caffeine/cellulose, the mixtures 7:3, 9:1 lie close to the neat caffeine. Analogously, the system with excess of cellulose activates the neurons in the proximity of that for pure cellulose. Another mixture, caffeine with glycine, exhibits a much different behavior (Figure 9c), thus, it is deemed incompatible. More mixtures were investigated in this manner, and the outcome of SOM were in agreement with other experimental techniques (DSC, FTIR). The proposed approach using SOMs outperforms the two other chemometric methods—the cluster analysis and the principal component analysis. Both these methods are not capable of resolving the mixtures adequately, when given the same characteristic temperatures as input.

## 6. Thermokinetic Analysis with Neural Networks

In this section, we discuss several applications of ANNs relevant to thermokinetic analysis. It should be immediately noted that some publications [17,91,92,93] on using ANNs for kinetic analysis deal with analysis of single-heating rate data or a single isothermal measurement. This approach is not recommended by the Kinetics Committee of the International Confederation for Thermal Analysis and Calorimetry (ICTAC) [28], as it does not allow for properly discriminating between the reaction models and, thus, for obtaining reliable kinetic parameters, as discussed further. Nevertheless, some of these works are discussed below to illustrate the development and current status of the ANNs applications in thermokinetics.

One of the benefits of the ANNs use for kinetic studies [9] is that they could bypass an explicit usage of Equation (1). This statement needs some clarification. For instance, some of the examples discussed in Section 3 show that one can predict the conversion degree as function of temperature and time without invoking Equation (1) and determining the kinetics triplets at all. However, such predictions are yet to be thoroughly verified to prove their general validity. On the other hand, evaluation of the kinetic triplets with the aid of ANN is possible without explicitly separating the k(T) and f(α) terms as well as making any assumptions about their mathematical form. Some evaluations of this type [21,94,95] have already been thoroughly tested in terms of predictions outside of the data space where the ANN is trained. However, such evaluations are based on the neural networks trained on the data simulated according to Equation (1), so that the knowledge of Equation (1) implicitly propagates into predictions.

Gauri et al. [15] compare the performance of the mechanistic kinetic modeling with the neural network. They consider a heterogeneous reaction of various limestones with SO_2_. Two reaction models have been proposed in literature for this type of reaction: the shrinking unreacted core model and the distributed pore model. As an example, we will consider only the simpler one, the shrinking unreacted core model:

(6) $$\frac{d\alpha }{dt}=\frac{3K_{s}}{\frac{1}{{\left(1-\alpha \right)}^{2/3}}+\gamma \left(\frac{1}{{\left(1-\alpha \right)}^{1/3}}-\frac{1}{{\left(1+P\alpha \right)}^{1/3}}\right)},$$

where $K_{s}=\left(k_{s}C_{s}\right)/\left(R_{0}N_{0}\right)$, $\gamma =\left(R_{0}k_{s}\right)/D_{e}$, $P=N_{0}\left(V_{p}-V_{r}\right)$ with *k*_s_ standing for the rate constant of surface reaction, *N*_0_—initial concentration of the solid reactant, *C_s_*—the surface concentration of gas, *R*_0_—the initial radius of the grain, *V_p_* and *V_r_*—the molar volume of solid product and reactant, *D_e_*—the diffusivity of Ca^2+^ ions. The more complex, distributed, pore model, additionally uses the pore properties that have to be evaluated in separate structural measurements. Obviously, the application of these mechanistic models is not straightforward. Moreover, there are some simplifying assumptions used in their derivation. On the contrary, the neural network model does not need these additional parameters and relies only on the TA data. Comparison of the fit performance by the two mechanistic models and by ANN shows that the latter is most accurate.

**Figure 10:**
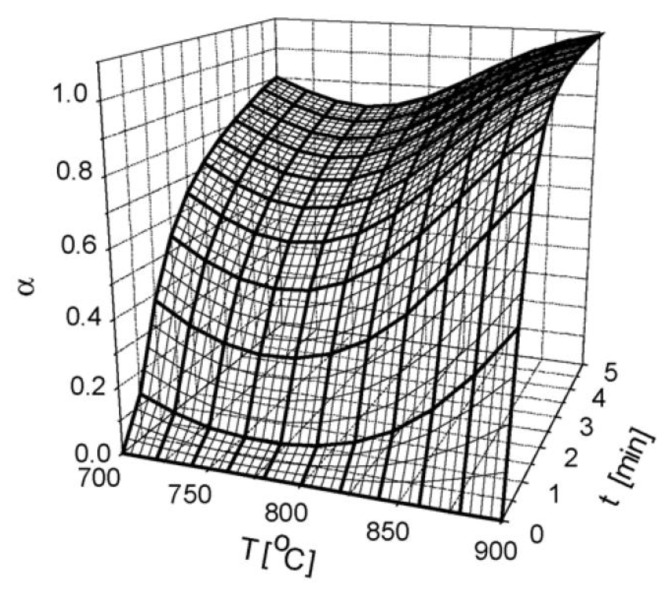
ANN (3-20-1) representation of the conversion degree for Fe_2_O_3_ reduction in 90%N_2_ + 5%CO + 5%H_2_ gas flow [19]. Reprinted with permission from Elsevier.

Another feature of using ANNs is noticed when analyzing a heterogeneous process of iron oxide reduction [19]. A complicating factor, along with a consecutive chemical reaction (Fe_2_O_3_ → Fe_3_O_4_ → FeO), is the structural changes of the interface throughout the reaction (sintering, cracking etc.). As a result, the conversion degree shows non-monotonic behavior, i.e., the reduction rate is delayed at temperatures about 750 °C (Figure 10). Authors note that this effect was found by other workers. The benefit of the ANN model is that it describes all the data, including this effect, whereas the single traditional mechanistic model fails to do that. Consideration of various reducing gas compositions shows that ANN also permits fitting the reaction course both in the kinetic and diffusion regimes. Accomplishing the same with the mechanistic models requires using two different models.

**Figure 11:**
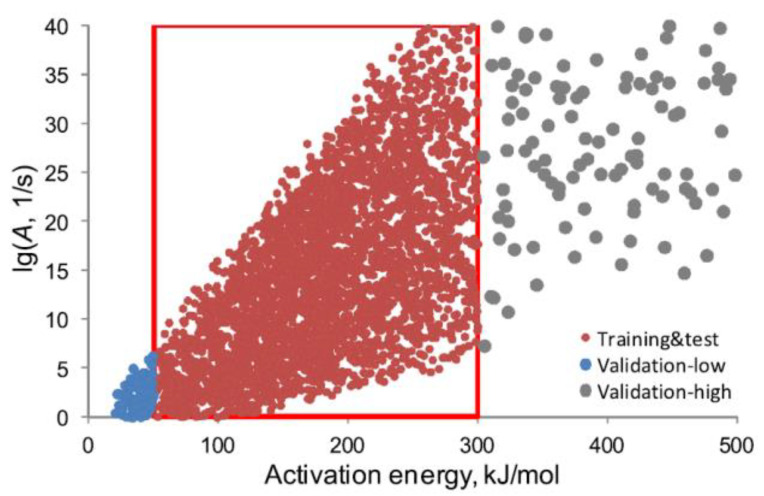
The plot populated with the (preexponential factor, activation energy) pairs used for training and testing of ANNs (red points), and validation of the results (gray and blue points) [21]. Reprinted with permission from Elsevier.

An idea of using the neural networks primarily for determination of the kinetic triplets has been put forward by Sbirrazzuoli et al. [9] and further developed by Conesa et al. [56], Muravyev and Pivkina [21], and other more recent workers [61,94,95,96,97]. As the approach develops, the complexity of the reaction domain has been increasing. The initiatory work [9] has employed for training and testing the second-order reaction models with activation energy 74–80 kJ mol^−1^, preexponential factor ln(*A*, s^−1^) = 18–20. Yet, the recent study [21] has considered ten reaction models with a large span of the activation energy and preexponential factor values (Figure 11).

**Figure 12:**
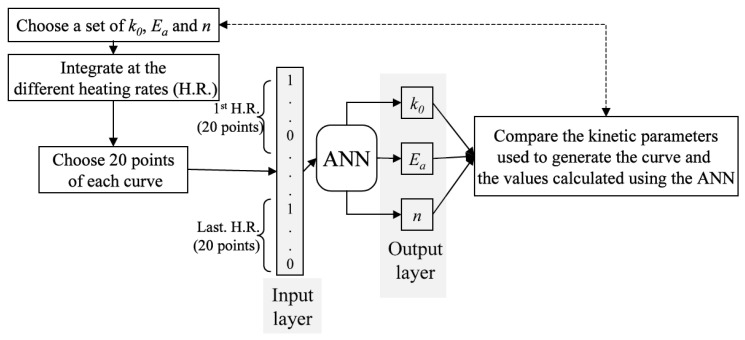
The framework of thermokinetic analysis with ANN proposed in Ref. [56]. Reprinted with permission from Elsevier.

The general idea of the approach is illustrated by Figure 12. The input vector is a set of sparse TA data (e.g., 20 points [56] or 49 points [21] for each run) at several heating rates. The output variables are the activation energy *E_a_*, preexponential factor *A*, and reaction order *n*. Conesa et al. [56] also employ ANN to evaluate the kinetic triplets when using the pyrolysis data of lignin, cellulose, and polyethylene as input. These triplets when inserted in Equation (1) give rise to the TGA data that match closely the experimental mass loss profiles. Thus, the neural network here is utilized as an alternative to traditional nonlinear optimization procedure.

Aghbashlo et al. [96,97] adopt the same approach but utilize a more advanced mathematical apparatus. The authors first use the principal component analysis to reduce the number of input variables from six (C, H, N, O, S content, and heating rate) to three. Then these transformed input data are fed into an adaptive neuro-fuzzy inference system (ANFIS) to produce the output kinetic parameters *E_a_*, *A*, *n*. ANFIS [98] represents a combination of neural network model with a fuzzy logic (FL) system. Additionally, the authors use the genetic algorithm (GA) to optimize the ANFIS parameters. The developed model results in the kinetic parameters that agree well with those obtained by traditional optimization procedure for five various biomass feedstocks (bamboo, *Phyllanthus emblica* kernel, *Musa balbisiana*, cattle manure, peanut shell). Although the computational approach from the study (ANN+FL+GA) is rather advanced, the major limitation is the constrained training that is limited to single heating rate and reaction order model.

Muravyev and Pivkina [21] extend the above-described approach [56] by considering a broad range of the activation energy and preexponential factor pairs (Figure 11). Additionally, instead of *n*-th order reaction model, 10 other reaction models are taken into account. Table 4 summarizes the results of their study. It is found that the best accuracy (determined as ANN performance out of the domain space used for training and testing, Figure 11) is achieved when the specific ANN is trained to determine only one component of the kinetic triplet (*E_a_*, *A*, f(α)). Additionally, it is shown that the suggested approach confirms some previously established facts: (1) failure of the kinetic analysis based on a single heating rate data [28]; (2) enhanced capability of controlled rate thermal analysis [99] to discriminate among the reaction models. Conceptually, since this approach involves the synthetic data as input, it paves the way to the reinforcement learning instead of the supervised learning for neural network. That is, one routine generates thermal analysis profiles for known kinetic parameters, while another (ANN) uses this large amount of data for training.

Huang et al. [94] have adopted the same approach, but further increased the number of reaction types to 17, and utilized a more advanced mathematical tool, the general regression neural network. Most importantly, the authors employ datasets of simulated nonisothermal experiments at several different heating rates. They stress that only at three heating rates one can achieve an acceptable recognition accuracy for the reaction model and that the accuracy improves significantly when the number of experiments (heating rates) used in ANN training is five. The relative error in the output preexponential factor and activation energy values is as low as 4% for the simulated data. Additionally, the authors applied the developed neural network to the experimental data for Li_4_Ti_5_O_12_/C high-temperature reaction. The ANN output agrees well with the literature results [100] of these data analysis accomplished with isoconversional and model-fitting kinetic approaches. It is noteworthy that according to the isoconversional analysis, the activation energy of the process is more or less constant throughout the reaction, thus, indicating the single-step reaction kinetics.

**Figure 13:**
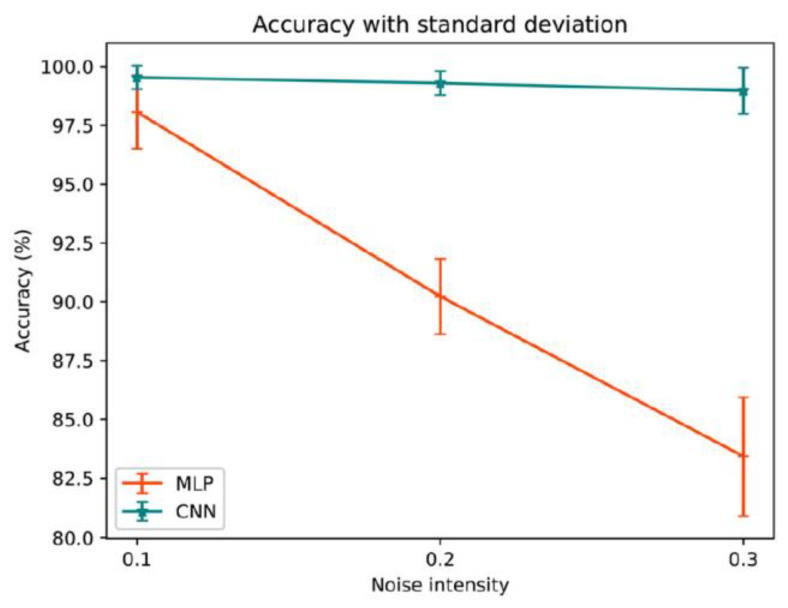
Comparison of the accuracy of convolutional neural network (CNN) and multilinear perceptron (MLP) models as a function of the introduced noise in the input data [95]. Reprinted with permission from Elsevier.

Kuang and Xu [95] have implemented the most advanced mathematical approach to this problem, i.e., the one-dimensional convolutional neural network (CNN). As in the previously considered work [94], the single neural network was utilized to estimate the whole kinetic triplet. The application of the trained CNN model for estimating the preexponential factor and activation energy has resulted in less than 3% error in case of using a dataset of three heating rates for training. Remarkably, the authors have used noisy input data to compare the CNN model with the one used in a previous study, the feedforward neural network (more specifically, multi linear perceptron, MLP). Figure 13 displays that with increasing the amplitude of the Gaussian noise, the performance of MLP starts decreasing, which does not apply to the CNN model. Obviously, the convolutional neural network shows superior robustness toward the noisy data. Finally, the model has been applied to the pine wood pyrolysis data. The details of calculations (e.g., the isoconversional plots) are not provided, but apparently all the discussed kinetic data are associated with the first mass loss stage (corresponds to ~85% of total mass loss). The kinetic parameters obtained with the CNN agree well with those computed by two isoconversional methods, Kissinger–Akahira–Sunose and Flynn–Wall–Ozawa methods.

It should be mentioned that ANN does not always yield the results that agree well with the isoconversional ones. These two approaches have been compared as applied to the kinetics in the system lumefantrine/molecularly imprinted polymer [101]. The isoconversional method has demonstrated that in the range of conversions from 0.05 to 0.90, the activation energy rises from roughly 80 to 150 kJ mol^−1^. This is a sure sign of the kinetics being complex, i.e., to involve at least two steps with significantly different activation energies [102]. Yet, the ANN estimates yield the activation energy whose values fluctuate in the range of approximately 160–200 kJ mol^−1^ depending on the heating rate used in analysis [101]. Of course, one cannot expect ANNs to reveal the multi-step nature of the process kinetic as long as the networks are trained on single-step kinetics. Likewise, the usage of single heating rate ANN analysis and training is a faulty approach to kinetic calculations as already demonstrated in a previous study [28].

**Figure 14:**
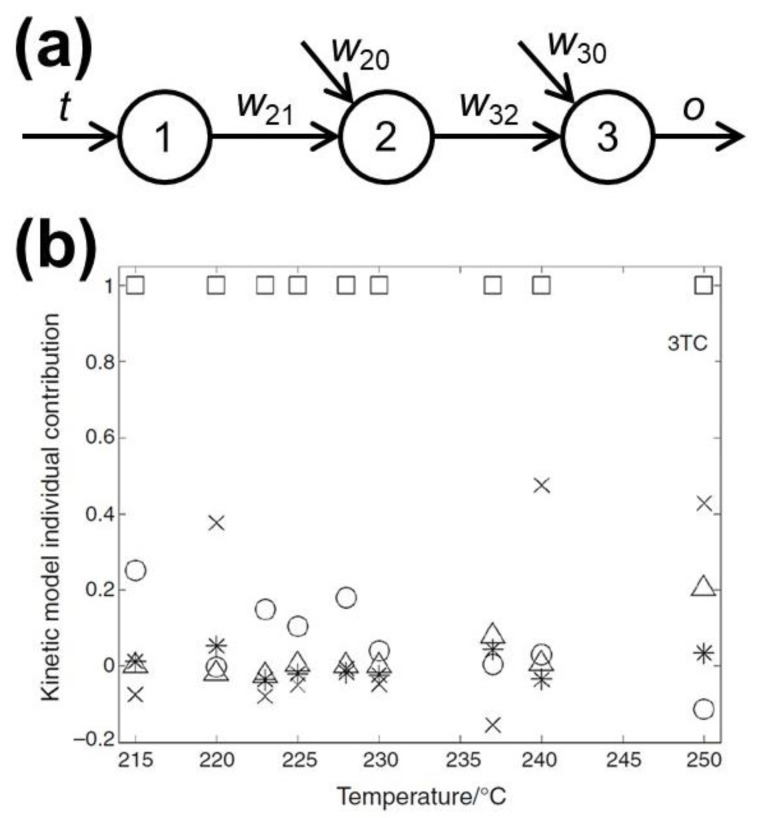
Kinetic analysis with ANNs comprising the activation functions for hidden layer neurons having the mathematical form of ideal reaction types. (**a**) The scheme of simple ANN model used in Ref. [18]. (**b**) Relative contributions of kinetic models in the course of thermal decomposition of lamivudine, squares depict three-dimensional diffusion model, crosses—linear contraction model, circles—second-order KJMAE nucleation-growth, triangles—Prout–Tompkins, asterisk—first-order reaction [93]. Reprinted by permission from Springer Nature.

A principally different attempt to perform kinetic analysis with ANNs has been made in a series of papers by Sebastiao and coworkers. The general idea is to utilize the neurons in the form of reaction model functions. The authors apply a similar approach for the description of isothermal processes of widely differing materials, i.e., rhodium (II) acetate [17,18], efavirenz and lamivudine [93], thalidomide [103], polyurethane [104], hybrid of carbon nanotubes with poly(3-hexylthiophene) [105], composite Li_4_SiO_4_/MgO [106], calcium trihydrate/furosemide system [107], and lumefantrine/molecularly imprinted polymer [101]. To show the basics of this method, we will follow the first paper [18], where the ANN with a single neuron in the hidden layer is considered (Figure 14). The neural network is fed with the time data from an isothermal measurement and outputs the conversion degree value. Given the weights and biases, as shown in Figure 13, and the sigmoidal activation function, the output value is:

(7) $$o=\frac{w_{32}}{1+e^{-\left(w_{21}i+w_{20}\right)}}+w_{30}.$$

Equation (7) can be compared with the best mechanistic kinetic model of Prout and Tompkins [108]:

(8) $$\frac{d\alpha }{dt}=k\alpha \left(1-\alpha \right)\to ln\frac{\alpha }{1-\alpha }=kt+C\to \alpha =\frac{1}{1+e^{-\left(kt+C\right)}}.$$

Using the similarity of the Equations (7) and (8), the logarithm of the optimized weight values *w*_21_ for each run is plotted against the reciprocal temperature to give the activation energy value. A larger number of the adjustable parameters in Equation (7) as compared to the traditional Equation (8) results in several times smaller residual error for the model.

The idea of using the activation functions to mimic the mechanistic reaction models is further advanced in another study by Sebastiao et al. [17]. More neurons have been added to the hidden layer, but their activation functions are set in an unusual, for ANNs, way. These activation functions have been selected to represent various kinetic models, e.g.:

(9) $${\left[-\ln\left(1-\alpha \right)\right]}^{1/m}=kt+C,$$

for Kolmogorov–Johnson–Mehl–Avrami–Erofeev nucleation-growth reaction [108], or:

(10) $$1-\frac{2\alpha }{3}-{\left(1-\alpha \right)}^{\frac{2}{3}}=kt+C,$$

for Ginstling–Brounshtein three-dimensional diffusion model [108]. That is, first the ideal reaction model (e.g., one of Equations (8)–(10)) is optimized on the experimental data, then the resulting parameters (*k*, *C*) are set as weight and bias for the respective neuron (e.g., as *w*_21_ and *w*_20_ for neuron designated as “2” in Figure 14a). Then, instead of the usual training of ANN using the back-propagation algorithm, the Levenberg–Marquardt optimization is performed with weights and biases for the hidden layer (i.e., *w*_21_ and *w*_20_) fixed, but adjusting the interconnection weights in the output layer (e.g., *w*_32_ in Figure 14a, note, that here no biases like *w*_30_ are considered for the outer neuron). From these weights the relative contribution of the individual reaction models to the whole process can be obtained. By way of example, Figure 14b represents such contributions for several isothermal experiments on the thermal decomposition of lamivudine [93]. Here, the three-dimensional diffusion model (Equation (8)) provides the main contribution to the process. Overall, the above discussed approach can be called as kinetic deconvolution by neural network.

As seen from above, the kinetic applications of ANN still are in their early stages of development. There remains much to be done for these applications to match the capabilities of the well-established isoconversional and model-fitting techniques [28,102]. In regard to this, there are two important principles that must be followed by any computational technique so that it can produce reliable kinetic triplets. First, in its computations, a technique must use simultaneously multiple heating rates or, more generally, multiple temperature programs [28]. For kinetic applications of ANN, it means that the network training sets must include data obtained at more than one heating rate. A failure to produce reliable kinetic triplets has already been demonstrated [21] for ANN trained on the data sets obtained at single heating rates. This problem is yet another manifestation of the fundamental failure of the kinetic techniques based on single heating rate [109]. In the case of the traditional isoconversional and model-fitting techniques, reliable kinetic evaluations require one to use simultaneously no less than 3–4 heating rates. The same principle should be adhered to when training and using ANNs for kinetic applications. Second, a computational technique must be capable of detecting and treating multi-step kinetics [102]. The reality of thermally stimulated processes in the condensed phase is such that they typically include more than one kinetic step. However, the literature analysis indicates that, so far, the ANNs used for kinetic purposes have been trained exclusively on single-step kinetics. Such restricted training limits drastically the application area of ANNs. To remove this important limitation, proper strategies for multi-step kinetics training ought to be developed. In the meantime, it seems prudent to support the usage of the ANNs trained on single-step kinetics by verifying whether the kinetics studied is actually a single-step one. The latter is readily confirmed as the absence of any significant variation in the isoconversional activation energy [102].

## 7. Other Applications Relevant to Thermal Analysis Studies

This section gives a short overview of papers that do not directly focus on thermal analysis data or thermokinetic parameters, but that can potentially find some applications in this field. First, neural networks are used to interpolate and extrapolate the temperature dependency of desired property. Thus, Wunderlich et al. [13] have proposed using ANNs for prediction of the heat capacity (c_p_) data. Note that this property is normally measured with the thermal analysis method of DSC, and it is of importance to interpolate and extrapolate the results to particular values of temperature. The experimental heat capacity values for 15 common polymers have been taken at every 10 K over the 110–360 K temperature range. These values serve as inputs for neural network (25-15-10), and the output was c_p_ at T = 10, 20, …, 100 K. The reported errors of trained ANN in the c_p_ data for five polymers from test set are less than 0.8%. Thus, the accuracy of this extrapolation procedure is better than typical experimental errors (1–5%). Another group of applications comprises predictions of thermal properties with ANNs. This topic is vast and is not considered here in detail. It worth mentioning, though, that artificial intelligence methods have been applied for prediction of thermal conductivity [110,111], vapor pressure [112], flammability [113], density [114], thermal stability [115], and mechanical properties [116]. Third group of applications includes some developments in experimental devices that can be potentially applied in future thermal analyzers and relevant instruments. For instance, Gao et al. [117] discuss an optimal iterative controller for nonlinear processes based on ANN model (note, that this topic has been widely studied, e.g., [118,119]). The authors demonstrate that the proposed system offers a better temperature control as compared to a standard PID controller. This method could be used in thermal analysis equipment where the temperature control is critical, e.g., in accelerating rate calorimeters [120].

## 8. Conclusions

Artificial neural networks (ANNs) are a system of powerful tools for solving data analysis problems in a broad variety of applications. Regarding the applications considered in the present review, they appear to be in an initiatory stage and use primarily the feed-forward neural networks as a computational tool. Beyond ANNs, some other artificial intelligence approaches have been sporadically applied to the TA problems and are worth mentioning. They include the expert analysis of thermogravimetric data [121], genetic approach [122] for determining kinetic parameters, modeling with adaptive neuro-fuzzy inference system [96,123,124] to predict the mass loss data, extreme gradient boosting algorithm [125] for product yield evaluation. A wider application of machine learning in these areas is expected as the computational tools become more readily available.

Our review indicates that the most popular ANN applications are associated with predicting pyrolysis product conversion. This popularity is driven by an obvious practical importance of the problem. Its solution requires carefully accounting for the TA data complexity via employing ANNs of an increasingly complex topology. In general, the performance of ANN models is quite good when assessed within the domain close to that used for the model development. However, evaluation of the performance outside such domain is almost absent in the literature. Studies on the extrapolation capabilities of the ANN models should certainly be encouraged.

Neural network models can also be applied to analyze the TA data directly as shown in examples of utilizing ANNs for identifying milk samples and probing compatibility of drug excipients. These applications can be readily extended to a large variety of multi-component materials, whose characteristic TA parameters change discretely with composition. Such applications are of obvious relevance to composition optimization and quality control. This type of usage of ANNs in assaying the TA data is definitely worthy of further exploration, especially in combination with some approaches from chemometrics, spectroscopy, and other fields such as filtering, baseline correction, deconvolution, classification, and so on.

In the area of the kinetic applications of ANNs, one should clearly differentiate between two groups of studies: (i) those that use single heating rate or single isothermal run for deriving the Arrhenius parameters; and (ii) those that derive the Arrhenius parameters via simultaneously using multiple heating rates or multiple temperature programs in general. The results of the first group must be treated with great caution because it is now established that single temperature program data are fundamentally incapable of yielding reliable kinetic parameters regardless of the computational technique used. A failure of such approach has already been demonstrated by comparing ANNs employing single and multiple heating rates. Only the multiple heating rates approach has proved to be correct, and this must be kept in mind while developing further kinetic applications of ANNs. Another crucial trait that must be implemented in future developments is the ability to recognize and treat the multi-step kinetics.

Overall, the ANN applications present a growing trend in the area covered by this review. ANN’s provide newer more sophisticated and flexible mathematical tools that are poised to accomplish a greater level of detail in the process description. Nevertheless, a greater level of detail does not necessarily translate to qualitatively deeper levels of understanding. Indeed, the latter are the true milestones of scientific progress. Only time will tell if ANNs spurred any considerable progress in the field under review. On the other hand, one must realize that no progress can ever be accomplished without trying new approaches and tools.

## Figures and Tables

**Table 1:** The list of the parameters used to model the coal pyrolysis, and some details on their incorporation to ANN [67]. Reprinted with permission from Elsevier.

Parameter	Format	Transformation	Input/Output
Particle holding time	Fuzzy class membership (s)	Logical (0/1)	Input
Particle holding time	Fuzzy class membership (min)	Logical (0/1)	Input
Freeboard residence time of volatiles	Numerical (s)	Linear, Inverse function, Inverse fourth root function, Fuzzy center (0) function	Input
Temperature	Numerical (°C)	Linear, Tanh, Square root function	Input
Time of preheating of particles	Numerical (s)	Linear, Inverse fourth root function, Fuzzy center (0) function, Inverse function	Input
Rate of heating	Numerical (K/s)	Linear, Inverse fourth root function, Inverse function	Input
Secondary reaction in freeboard	Qualitative (none, small, medium, great)	Enumerated strings: small, medium, great	Input
Secondary reaction in bed	Qualitative (small, medium, great)	Enumerated strings: small, great	Input
Reactor type	String (flb, spb, hr, wm) ^1^	Enumerated strings: flb, spb, hr, wm	Input
Particle size	Qualitative (fine, coarse)	Enumerated strings: fine, coarse	Input
Concentration of coal particles	Qualitative (low, medium, high)	Enumerated strings: low, medium, high	Input
Bed depth	Qualitative (shallow, deep, medium, mono-layer, mono-layer-shallow)	Enumerated strings: shallow, deep, medium, mono-layer, mono-layer-shallow	Input
Sample size	Numerical (g of coal)	Linear, Inverse fourth root function, Inverse function	Input
Coal: wt% ash (d.b.) ^2^	Numerical (% db)	Linear, Inverse fourth root function, Inverse function	Input
Coal: wt% volatiles (d.a.f.) ^3^	Numerical (% daf)	Linear, Fourth power function, Exponential	Input
Carrier gas velocity	Numerical (m/s)	Linear, Inverse fourth root function, Inverse function	Input
Yield of tar	Numerical (wt%)	Linear	Output
Yield of volatiles	Numerical (wt%)	Inverse function	Output
Yield of char	Numerical (wt%)	Inverse function	Output

^1^ Designation of reactor types: flb = fluidized bed reactor, spb = spouted bed reactor, hr = hot-rod fixed bed reactor, wm = wire-mesh reactor. ^2^ d.b.—dry basis value (except all moisture). ^3^ d.a.f.—dry ash free value (except all moisture and ash).

**Table 2:** Details of the neural network models for prediction of various thermal signals for poly(ethylene oxide)/clay nanocomposites [82]. Reprinted with permission from Elsevier.

ANN and Training Parameters	DMA	DMA	TGA	DSC
Input data	Temperature and clay wt%	Temperature and clay wt%	Temperature and clay wt%	Temperature and clay wt%
Output data	E’	tanδ (E”/E’)	Weight loss	Heat flow
Training data	193	193	406	565
Testing data	47	47	45	141
Number of hidden layers	1	1	2	2
Activation functions	Tansig-Purelin	Tansig-Purelin	Tansig-Logsig-Purelin	Tansig-Logsig-Purelin
Number of neurons in hidden layer	9	9	4–3	5–4
Number of iterations	288	152	48	65
Regression (R2)	0.9997	0.9982	0.99994	0.99985

**Table 3:** Thermal properties derived from the DSC data for various milk samples [40]. Reprinted with permission from Elsevier.

Parameters	Control	Starch1	Starch2	Starch3	Starch4	Starch5
*T*_oc_ (°C)	−0.430	0.035	0.065	−0.480	−0.670	−0.535
*T*_pc_ (°C)	−0.400	−0.290	−0.035	−0.795	−0.535	−0.740
Δ*H*_c_ (J g^−1^)	−253.50	−233.430	−247.197	−244.578	−243.339	−244.377
*T*_om_ (°C)	−0.385	−0.645	−0.535	−1.140	−1.925	−1.230
*T*_pm_ (°C)	5.315	4.600	5.665	5.495	5.350	6.510
Δ*H*_m_ (J g^−1^)	220.833	263.704	236.707	199.669	217.466	226.643
*T*_ob_ (°C)	104.505	103.955	105.645	105.400	107.025	163.480
*T*_pb_ (°C)	123.330	118.615	131.570	123.805	121.935	168.880
Δ*H*_b_ (J g^−1^)	3577.5	870.990	4313.429	1416.971	1675.230	844.814

Notes: The results show only the mean value, the standard deviation values can be found in the original paper. Control sample = pure milk without additives, samples called Starch 1, 2, 3, 4, 5 = 1, 2, 3, 4, 5 g L^−1^ milk. Subscripts 0 and p denote the onset and peak values, subscripts *c*, *m*, and *b* correspond to crystallization, melting, and boiling processes.

**Table 4:** ANN details and performance of models developed for kinetic analysis in Ref. [21]. Reprinted with permission from Elsevier.

#	Input		Output		Cases	*N_h_*	*P*, %		*AC*, %
	Parameters	*N_i_*	Parameters	*N_o_*			Training	Test	
1	*T(α)* B = 5 K/min	49	Reaction model	6	3278	15	99.1	98.2	88
2	*T(α)* B = 5 K/min	49	*E_a_*	1	6106	32	93.8	92.2	62
3	*T(α)* β = 0.5, 20 K/min	98	Reaction model	6	199	18	99.8	99.4	100
4	*T(α)* β = 0.5, 20 K/min	98	*E_a_*	1	147	14	99.9	99.9	98
5	*T(α)* β = 0.5, 20 K/min	98	lg*A*	1	139	10	99.9	99.9	98
6	*T(α)* β = 0.5, 20 K/min	98	*E_a_*, lg*A*	2	147	13	99.9	99.9	98(E), 92(A)
7 ^1^	*T(α)* β = 0.5, 20 K/min	50	Reaction model	10	1109	20	99.8	99.4	100
8 ^1^	*T(α)* β = 0.5, 5, 20 K/min	75	Reaction model	10	483	25	99.8	99.1	100
9	*T(α) c* = 1∙10^−3^ min^−1^	49	Reaction model	6	851	27	90.1	84.3	65
10 ^2^	*T(α) c* = 1∙10^−3^ min^−1^	49	Reaction model	6	678	15	99.9	99.9	100
11 ^2^	*T(α) c* = 1∙10^−3^ min^−1^	49	Reaction model, *E_a_*, lg*A*	8	678	21	99.6	99.2	81 (E), 44(A), 100 (R)

Notes: *N*_i_, *N*_h_ and *N*_o_ are the number of neurons in input, hidden, and output layer of network, *P* and *AC*—the performance and accuracy of the respective ANN. ^1^ For these models, the reaction types (B1, D2, D3, F2, L2, F1, P2, R3, A2, A3) were used, and the step for conversion degree Δα = 0.05 was applied instead of Δα = 0.02. ^2^ For these models, the reaction types (F1, A2, A3, D1, D3, B1) were used instead of (F1, R2, A3, F2, D3, B1).

## Data Availability

Not applicable.
